# Assessment of Quality of Life of Transgender and Gender-Diverse Children and Adolescents in Melbourne, Australia, 2017-2020

**DOI:** 10.1001/jamanetworkopen.2022.54292

**Published:** 2023-02-02

**Authors:** Lidia Engel, Ishani Majmudar, Cathrine Mihalopoulos, Michelle A. Tollit, Ken C. Pang

**Affiliations:** 1 School of Public Health and Preventive Medicine, Monash University, Melbourne, Victoria, Australia; 2 Deakin Health Economics, Deakin University, Burwood, Victoria, Australia; 3 The Royal Children’s Hospital, Parkville, Victoria, Australia; 4 Murdoch Children’s Research Institute, Parkville, Victoria, Australia; 5 Department of Paediatrics, The University of Melbourne, Parkville, Victoria, Australia

## Abstract

**Importance:**

Transgender and gender-diverse (TGD) children and adolescents may experience not only gender dysphoria but also depression and anxiety, all of which are likely to be associated with reduced quality of life (QOL). Despite this, little is known about QOL in this population.

**Objectives:**

To identify demographic, social, and clinical characteristics associated with reduced QOL in TGD children and adolescents; compare their QOL with age-matched population-based norms and that of young people with common mental health problems; and evaluate the association between gender dysphoria and QOL.

**Design, Setting, and Participants:**

In this cohort study, baseline data were derived from questionnaires completed in a prospective cohort study (Trans20) of TGD children aged 6 to 12 years and adolescents aged 13 to 17 years first seen at the Royal Children’s Hospital Gender Service (Melbourne, Australia) between February 2017 and February 2020.

**Main Outcomes and Measures:**

The main outcome was QOL, measured using the Child Health Utility 9D instrument (CHU-9D). Data collection included demographic information, social factors (eg, bullying, lack of support, and social transition), and clinical characteristics (eg, gender identity, gender dysphoria, and mental health difficulties). Population norms and CHU-9D data for Australian youths with mental health diagnoses were derived from published literature.

**Results:**

The TGD cohort comprised 525 children and adolescents aged 6 to 17 years (median age, 14 years [IQR, 12-16 years]; 364 [69.33%] presumed female at birth). The mean (SD) CHU-9D score was 0.46 (0.26). Compared with population norms, TGD children (0.58 [0.27] vs 0.81 [0.16]; *P* < .001) and adolescents (0.41 [0.25] vs 0.80 [0.14]; *P* < .001) had significantly lower scores. Within the TGD cohort, mean (SD) scores were significantly lower in adolescents (0.41 [0.24] vs 0.62 [0.25]; *P* < .001), those assigned female at birth (0.43 [0.26] vs 0.55 [0.25]; *P* < .001), those reporting mental health problems (0.37 [0.23] vs 0.57 [0.25]; *P* < .001) and physical health problems (0.41 [0.26] vs 0.48 [0.26]; *P* = .04), and those who were bullied (0.38 [0.24] vs 0.52 [0.25]; *P* < .001). Gender dysphoria alone was associated with a lower mean (SD) CHU-9D score (0.51 [0.23]) than that in control adolescents with serious mental health conditions such as depression (0.64 [0.26]) and anxiety (0.70 [0.24]) and was an independent factor associated with QOL.

**Conclusions and Relevance:**

In this cohort study of TGD children and adolescents in Australia, QOL was worse in this population than in age-matched, population-based peers. Quality of life associated with gender dysphoria was substantially worse than that seen in young people with common mental health conditions. These findings emphasize the risk of poor QOL among TGD young people and the need to better support them.

## Key Points

**Question:**

What is the quality of life (QOL) of transgender and gender-diverse children and adolescents compared with their age-matched peers?

**Findings:**

This cohort study of 525 transgender children and adolescents in Australia revealed that their QOL scores were substantially lower than age-matched population norms. Gender dysphoria in this cohort was associated with a lower QOL score than that for control adolescents with common mental health problems (ie, anxiety and depression).

**Meaning:**

The findings suggest that gender dysphoria is associated with worse QOL in transgender children and adolescents, emphasizing the need for society and health services to better support these young people.

## Teaser

This cohort study compares the quality of life of transgender and gender-diverse children and adolescents in Australia with that of their age-matched peers.

## Introduction

Transgender and gender-diverse (TGD) individuals have a gender identity that differs from the gender that was presumed for them at birth.^1^ The number of TGD people worldwide is estimated at 25 million,^1^ and the proportion of young people who are TGD ranges from 1.2% to 2.7%.^2,3,4^ Transgender and gender-diverse young people are at risk of bullying, physical assault, discrimination, and social exclusion, all of which contribute to poor mental health.^5^ Consistent with this, TGD young people have high rates of depression, anxiety, self-harm, and suicide.^2,5,6,7,8^ In addition, many TGD young people experience gender dysphoria, which is the distress that arises when a person’s presumed gender at birth does not match their gender identity.^9^ To help alleviate this dysphoria, increasing numbers of TGD young people have been referred to specialist pediatric gender clinics in recent years.^6,7^

While many studies have documented poor mental health among TGD young people, few have assessed their overall quality of life (QOL). Quality of life is a multidimensional construct that encompasses physical, emotional, mental, social, and behavioral components of well-being and functioning as perceived by individuals.^10^ While a recent literature review synthesized QOL outcomes in transgender adults and observed that transgender people had worse QOL than the general population,^11^ only a handful of studies to date have examined QOL in TGD children and adolescents.^12,13,14,15,16^ Some of these studies reported that QOL was worse in TGD children and adolescents compared with population norms in the US and Germany and with individuals with chronic medical conditions, such as diabetes, asthma, or cancer.^13,14,16^ Other studies examined specific factors that might impact QOL in TGD children and adolescents and observed that while parental support was associated with improved QOL,^15^ it is likely that autism spectrum disorder was associated with worse QOL.^17^

Existing studies that examined QOL in TGD children and adolescents are limited in a number of important ways. First, they have been based on small sample sizes ranging from only 55 to 142 participants. Second, they have predominantly focused on adolescents and have rarely included children. Third, it remains unclear whether gender dysphoria is associated with worse QOL once other mental health problems are taken into account. Finally, to our knowledge, no studies have sought to undertake a comprehensive examination of how various demographic, social, and clinical characteristics might be associated with QOL in TGD children and adolescents. For example, it is unclear whether and to what extent age, different gender identities, bullying, and lack of support for one’s gender diversity are associated with differences in QOL. Similarly, it is unknown whether social transition, which includes coming out to others, using different names and pronouns, and changing one’s appearance, is associated with any change in QOL. Using baseline data from a large clinical cohort of TGD children and adolescents, this study aimed to address these critical knowledge gaps. The objectives of this study were to (1) identify demographic, social, and clinical characteristics associated with reduced QOL in TGD children and adolescents; (2) compare QOL in TGD children and adolescents with age-matched population-based norms and with that in young people with common mental health problems; and (3) evaluate the association between gender dysphoria and QOL among TGD children and adolescents.

## Methods

The Royal Children’s Hospital Human Research Ethics Committee approved this cohort study; informed consent was not required because data were collected as standard clinical care.^18^ In reporting the results of our study, we adhered to the Strengthening the Reporting of Observational Studies in Epidemiology (STROBE) reporting guideline.

### TGD Sample

Data for TGD children and adolescents were drawn from a prospective, longitudinal cohort study known as Trans20 conducted at the Royal Children’s Hospital Gender Service (RCHGS) in Melbourne, Australia.^18^ Young people who first attended the RCHGS between February 2017 and February 2020 and a nominated parent or primary caregiver were asked to complete an online questionnaire prior to their first appointment. The resultant baseline data form the basis of the current study, and only young people who attended an initial appointment, spoke English, and had at least 1 completed baseline questionnaire were included. Relevant instruments used in this study to assess demographic, social, and clinical characteristics are summarized in the eTable in Supplement 1. Questionnaires were completed by patients and parents as part of routine clinical care at RCHGS. This information was analyzed under a clinical audit framework.

### Quality of Life

Quality of life was assessed using the Child Health Utility 9D instrument (CHU-9D),^19,20,21^ a preference-based health-related QOL instrument. The CHU-9D was originally designed for children aged 7 to 11 years, but subsequent research has demonstrated its validity in adolescents up to 17 years of age^22^; it has been commonly used in health economic evaluations across many different contexts and has support for its validity.^20,22,23,24,25,26,27,28,29^ The measure contains 9 dimensions (worried, sad, pain, tired, annoyed, schoolwork or homework, sleep, daily routine, and activities). Each dimension has 5 response levels, in which higher levels represent greater problems. The scoring of the CHU-9D for this study was based on the Australian tariff, which represents preferences of 1982 Australian adolescents aged 11 to 17 years about the health states defined by the CHU-9D measure.^30^ The resulting health utility scores were anchored on a 0 (dead) to 1 (full health) scale, with negative scores denoting states considered worse than being dead.

### Demographic Information

Demographic characteristics were collected via a young person’s and/or parent’s reported questionnaire, which included information on age, gender presumed at birth, educational level, and country of birth. We dichotomized the sample into childhood and adolescence using age 12 years as a cutoff, given the important developmental and social milestones occurring around this time (eg, time of transition to high school). Socio-Economic Indexes for Areas were derived based on the geographical area of the participant,^31^ from which the Index of Relative Socio-Economic Disadvantage was derived.

### Clinical Characteristics

Young TGD people were asked to describe their current gender identity, which was subsequently categorized as either binary (subdivided into transgender masculine and transgender feminine), nonbinary, or unsure.^32^ Gender dysphoria was assessed using the Gender Preoccupation and Stability Questionnaire (GPSQ),^33^ for which a total score of 28 or more corresponds to clinically significant gender dysphoria. While the GPSQ contains questions that assess the stability of gender identity, it was specifically designed to assess gender dysphoria and, related to this, has been reported to be “an effective, valid, reliable outcome tool to measure gender dysphoria.”^33^ To assess young people’s general health, parents were asked to report whether their child had any preexisting mental or physical health conditions. Mental health was also assessed using the parent-reported Child Behavior Checklist (CBCL), which describes a child’s behavior and functioning during the previous 6 months and contains 6 norm-referenced *Diagnostic and Statistical Manual of Mental Disorders, Fifth Edition*–oriented scales that include depressive problems, anxiety problems, somatic problems, attention-deficit/hyperactivity problems, oppositional defiant problems, and conduct problems.^34,35^ All CBCL scales have a T score of 50 and an SD of 10; T scores of 70 or higher were considered in the clinical range as previously described.^35^ Suicidality was assessed using the Columbia Suicide Severity Rating Scale,^36^ an instrument designed to quantify the severity of suicidal ideation and behavior. It was administered to people aged 12 years or older by a clinician during the first visit, and scores were dichotomized into suicidality present and suicidality not present. The presence of a clinician ensured that relevant follow-up was provided to the young person if they were identified as being at risk of suicide.

### Social Factors

The Gatehouse Bullying Scale was used to assess whether the young person had been bullied.^37^ This instrument comprises 12 items assessing types of bullying. Participants were classified as being bullied if they responded to having experienced 1 or more bullying acts. The extent to which the young person had undertaken a social gender transition was assessed using the Social Transition Questionnaire developed by the Trans20 team.^38^ This tool was used to ask respondents to report the extent to which they had transitioned with respect to their name, pronouns, and appearance across different settings (home, school, and online). Young persons’ responses were categorized into fully transitioned, partially transitioned, and not transitioned. Finally, the young person was also asked to report the level of support they received in relation to their gender identity from their mother, father, siblings, teachers, and friends, and responses were categorized into fully supportive or not.

### Population Norm Samples

CHU-9D utility scores from the TGD sample were compared with population norms reported for Australian adolescents. Three published control samples were used: (1) a community-based sample of 2020 adolescents aged 11 to 17 years who were recruited via an online panel^24^; (2) a sample from the population-based cross-sectional study of the Longitudinal Study of Australian Children that included 1853 children aged 11 to 12 years^39^; and (3) a sample from a recent population-based survey of the Young Minds Matter (YMM) study comprising 2967 adolescents aged 11 to 17 years.^40^ The latter also included adolescents reporting mental health conditions, including attention-deficit/hyperactivity disorder, major depressive disorder, anxiety disorder, and conduct disorder.

### Statistical Analysis

Descriptive statistics were used to summarize participant characteristics, including percentages for categorical data and means and SDs for continuous data, for the total sample and by age cohort. Distribution of responses to the 9 CHU-9D dimensions were graphically represented in a stacked frequency histogram. A centile chart was used to visualize CHU-9D scores by age and gender presumed at birth. Nonparametric tests were performed to compare the CHU-9D scores across subgroups, with the Wilcoxon rank sum test used for dichotomous groups and the Kruskal-Wallis test for more than 2 groups (post hoc analyses for multiple comparisons of groups were based on the Dunn test). Effect sizes were calculated using Cohen *d*, with values of 0.2, 0.5, and 0.8 or greater interpreted as representing small, moderate, or large effects, respectively.^41^ Whether gender dysphoria was associated with QOL independently of other mental health conditions was assessed using ordinary least-squares regression analysis. Thereby, presence of gender dysphoria was included as the independent variable and was adjusted for age, gender presumed at birth, and mental health problems. When comparing the TGD sample with the control samples, the TGD sample was matched to the respective age range reported in the population norms and CHU-9D scores were compared using *t* tests for 2 independent samples. Complete data were used for the analyses, and listwise deletion was used for handling missing values. Two-sided *P* < .05 was the level of statistical significance used for all analyses. All analyses were undertaken in Stata, version 15 (StataCorp LLC).^42^

## Results

### TGD Sample Characteristics

In total, baseline data from 525 TGD children and adolescents from Trans20 were available for analysis. Characteristics of the study participants are described in Table 1. The median age was 14 years (IQR, 12-16 years), 364 (69.33%) were presumed female and 161 (30.67%) were presumed male at birth, the majority (484 [92.19%]) were born in Australia, and there was an underrepresentation of individuals from more socioeconomically disadvantaged areas. In terms of gender identity, 281 (53.52%) reported a transgender masculine identity, 105 (20.00%) a transgender feminine identity, and 66 (12.57%) a nonbinary gender identity; 67 (12.76%) were unsure.

**Table 1.** Characteristics of Transgender and Gender-Diverse Children and Adolescents Included in the Study

Characteristic	Participants, No. (%)		
	Total (N = 525)	Aged 6-12 y (n = 142)	Aged 13-17 y (n = 383)
Age, median (IQR), y	14 (12-16)	10 (7-11)	15 (14-16)
Gender presumed at birth			
Female	364 (69.33)	71 (50.00)	293 (76.50)
Male	161 (30.67)	71 (50.00)	90 (23.50)
Grade in school			
Elementary or lower	125 (23.81)	125 (88.03)	0
Secondary	394 (75.05)	16 (11.27)	378 (98.69)
Other	6 (1.14)	1 (0.70)	5 (1.31)
Country of birth			
Australia	484 (92.19)	131 (92.25)	353 (92.17)
Other	38 (7.24)	8 (5.63)	30 (7.83)
Missing	3 (0.57)	3 (2.11)	0
SEIFA-IRSD, quintile^a^			
1	47 (8.95)	12 (8.45)	35 (9.14)
2	80 (15.24)	23 (16.20)	57 (14.88)
3	100 (19.05)	15 (10.56)	85 (22.19)
4	131 (24.95)	44 (30.99)	87 (22.72)
5	159 (30.29)	44 (30.99)	115 (30.03)
Missing	8 (1.52)	4 (2.82)	4 (1.04)
Gender identity			
Transgender			
Masculine identity	281 (53.52)	61 (42.96)	220 (57.44)
Feminine identity	105 (20.00)	38 (26.76)	67 (17.49)
Nonbinary	66 (12.57)	19 (13.38)	47 (12.27)
Unsure	67 (12.76)	23 (16.20)	44 (11.49)
Prefer not to answer	6 (1.14)	1 (0.70)	5 (1.31)
Mental health problem^b^			
Anxiety	173 (32.95)	41 (28.87)	132 (34.46)
Depression	212 (40.38)	39 (27.46)	173 (45.17)
Somatic problems	70 (13.33)	11 (7.75)	59 (15.40)
Conduct disorder	36 (6.86)	17 (11.97)	19 (4.96)
Oppositional defiant problems	48 (9.14)	14 (9.86)	34 (8.88)
ADHD	34 (6.48)	12 (8.45)	22 (5.74)

Abbreviations: ADHD, attention-deficit/hyperactivity disorder; IRSD, Index of Relative Socio-Economic Disadvantage; SEIFA, Socio-Economic Indexes for Areas.

^a^ Quintile 1 indicates most disadvantaged.

^b^ Based on the Child Behavior Checklist. Young people could have multiple mental health problems.

### QOL of TGD Children and Adolescents

The mean (SD) CHU-9D utility score for the total TGD cohort was 0.46 (0.26). The Figure shows the distribution of responses across the different CHU-9D dimensions and notable impairments in the following domains: feeling worried, sad, or tired; having sleep problems; and having impairments in joining activities. Subgroup analyses of CHU-9D scores following stratification based on various demographic, clinical, and social characteristics are shown in Table 2. Mean (SD) CHU-9D scores were significantly lower for adolescents aged 13 to 17 years (0.41 [0.24]) compared with children aged 6 to 12 years (0.62 [0.25]) (Cohen *d*, 0.84; *P* < .001) (eFigures 1 and 3 in Supplement 1) and were also significantly lower for those assigned female at birth (0.43 [0.26]) compared with those assigned male at birth (0.55 [0.25]) (Cohen *d*, 0.49; *P* < .001), especially in those aged 13 to 17 years (eFigures 2 and 3 in Supplement 1). Transgender masculine individuals (0.44 [0.27]) had a similar mean (SD) CHU-9D score to those with a nonbinary identity (0.44 [0.25]) but a lower score compared with transgender feminine individuals (0.52 [0.26]) or those unsure of their gender identity (0.50 [0.24]). For the nonbinary participants assigned female at birth, the mean (SD) CHU-9D score was lower (0.38 [0.25]) compared with that among nonbinary participants assigned male at birth (0.57 [0.20]). However, the difference was only statistically significant for the cohort aged 6 to 12 years. The majority of TGD individuals who completed the gender dysphoria questionnaire (administered only to those aged 11 years or older) experienced clinically significant gender dysphoria (389 of 408 [95.34%]) and had a lower mean (SD) CHU-9D score than did those who did not have clinically significant gender dysphoria (0.40 [0.24] vs 0.58 [0.27]; Cohen *d*, 0.72; *P* = .008). Those with mental health difficulties based on the CBCL had a lower mean (SD) score than did those without mental health difficulties (0.37 [0.23] vs 0.57 [0.25]; Cohen *d*, 0.85; *P* < .001), as did those at risk of suicide (0.27 [0.21] vs 0.43 [0.24]; Cohen *d*, 0.67; *P* < .001). The mean (SD) CHU-9D score was also lower in those who reported physical health problems (0.41 [0.26] vs 0.48 [0.26]; Cohen *d*, 0.25; *P* = .04). Being bullied was also associated with lower mean (SD) score (0.38 [0.24] vs 0.52 [0.25]; Cohen *d*, 0.56; *P* < .001), especially in those aged 13 to 17 years. In terms of social transition, those who had fully socially transitioned had lower CHU-9D scores; however, this was only significant in the adolescent cohort and not in those aged 6 to 12 years. No statistically significant differences were observed based on the level of support received from a mother, father, friends, and teachers, although slightly lower scores were reported for those having no support from each of these people. Only those reporting full support from siblings had a significantly better QOL compared with those whose siblings were not supportive (mean [SD] CHU-9D score, 0.49 [0.27] vs 0.41 [0.26]; Cohen *d*, −0.31; *P* = .04).

**Figure.**
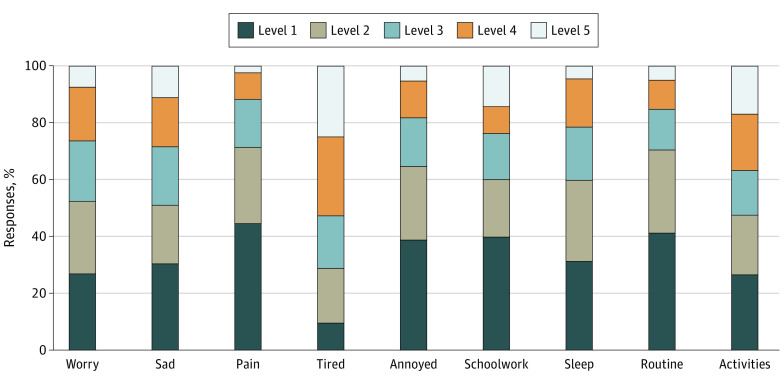
Distribution of Responses to the Child Health Utility 9D Instrument Dimensions Each of the 9 dimensions has 5 severity levels, with higher levels indicating greater problems.

**Table 2.** CHU-9D Scores by Participant Characteristics

Characteristic	Participants											
	Total (N = 525)				Aged 6-12 y (n = 142)				Aged 13-17 y (n = 383)			
	No./total No. (%)^a^	CHU-9D score, mean (SD)	*P* value	Effect size^b^	No./total No. (%)^a^	CHU-9D score, mean (SD)	*P* value	Effect size^b^	No./total No. (%)^a^	CHU-9D score, mean (SD)	*P* value	Effect size^b^
Total sample	525 (100)	0.46 (0.26)	NA	NA	142/525 (27.05)	0.62 (0.25)	NA	NA	383/525 (72.95)	0.41 (0.24)	NA	NA
Age, y												
6-12	142/525 (27.05)	0.62 (0.25)	<.001	0.84	NA	NA	NA	NA	NA	NA	NA	NA
13-17	383/525 (72.95)	0.41 (0.24)			NA	NA			NA	NA		
Gender presumed at birth												
Female	364/525 (69.33)	0.43 (0.26)	<.001	0.49	71/142 (50.00)	0.58 (0.28)	.12	0.30	293/383 (76.50)	0.39 (0.24)	.004	0.34
Male	161/525 (30.67)	0.55 (0.25)			71/142 (50.00)	0.65 (0.23)			90/383 (23.50)	0.47 (0.23)		
Gender identity												
Nonbinary	66/519 (12.72)	0.44 (0.25)	.04^c^	0.02	19/141 (13.48)	0.57 (0.25)	.87^d^	0.01	47/378 (12.43)	0.38 (0.24)	.14^e^	0.01
Transgender												
Masculine	281/519 (54.14)	0.44 (0.27)			61/141 (43.26)	0.63 (0.27)			220/378 (58.20)	0.39 (0.24)		
Feminine	105/519 (20.23)	0.52 (0.26)			38/141 (26.95)	0.62 (0.28)			67/378 (17.72)	0.46 (0.24)		
Unsure	67/519 (12.91)	0.50 (0.24)			23/141 (16.31)	0.63 (0.19)			44/378 (11.64)	0.43 (0.24)		
Nonbinary gender identity												
Assigned female at birth	46/66 (69.70)	0.38 (0.25)	.003	−0.76	7/19 (36.84)	0.41 (0.30)	.02	−1.16	39/47 (82.98)	0.38 (0.25)	.48	−0.19
Assigned male at birth	20/66 (30.30)	0.57 (0.20)			12/19 (63.16)	0.67 (0.16)			8/47 (17.02)	0.42 (0.17)		
**Clinical factors**												
Gender dysphoria score^f^												
<28	19/408 (4.66)	0.58 (0.27)	.008	0.72	5/25 (20.00)	0.64 (0.28)	.09	0.93	14/383 (3.66)	0.56 (0.28)	.05	0.63
≥28	389/408 (95.34)	0.40 (0.24)			20/25 (80.00)	0.40 (0.25)			369/383 (96.34)	0.40 (0.24)		
Suicidality^g^												
Present	342/387 (88.37)	0.43 (0.24)	<.001	0.67	24/25 (96.00)	0.48 (0.29)	.21	NA	318/362 (87.85)	0.43 (0.24)	<.001	0.65
Not present	45/387 (11.63)	0.27 (0.21)			1/25 (4.00)	0.14 (0)			44/362 (12.15)	0.28 (0.21)		
Parent-reported mental health problems												
No	265/518 (51.16)	0.53 (0.26)	<.001	0.52	95/142 (66.90)	0.64 (0.25)	.07	0.33	170/376 (45.21)	0.47 (0.24)	<.001	0.45
Yes	253/518 (48.84)	0.40 (0.25)			47/142 (33.10)	0.56 (0.26)			206/376 (54.79)	0.36 (0.24)		
Parent-reported physical health problems												
No	442/518 (85.33)	0.48 (0.26)	.04	0.25	125/142 (88.03)	0.63 (0.25)	.08	0.46	317/376 (84.31)	0.42 (0.24)	.30	0.14
Yes	76/518 (14.67)	0.41 (0.26)			17/142 (11.97)	0.51 (0.27)			59/376 (15.69)	0.38 (0.25)		
Mental health problems^h^												
No	248/513 (48.34)	0.57 (0.25)	<.001	0.85	78/140 (55.71)	0.69 (0.24)	<.001	0.78	170/373 (45.58)	0.52 (0.24)	<.001	0.87
Yes	265/513 (51.66)	0.37 (0.23)			62/140 (44.29)	0.51 (0.24)			203/373 (54.42)	0.32 (0.21)		
Depressive problems												
No	301/513 (58.67)	0.56 (0.25)	<.001	0.96	101/140 (72.14)	0.66 (0.24)	.001	0.68	200/373 (53.62)	0.51 (0.23)	<.001	0.98
Yes	212/513 (41.33)	0.33 (0.22)			39/140 (27.86)	0.49 (0.24)			173/373 (46.38)	0.30 (0.20)		
Anxiety problems												
No	340/513 (66.28)	0.52 (0.26)	<.001	0.63	99/140 (70.71)	0.64 (0.25)	.01	0.47	241/373 (64.61)	0.47 (0.24)	<.001	0.69
Yes	173/513 (33.72)	0.36 (0.23)			41/140 (29.29)	0.53 (0.23)			132/373 (35.39)	0.31 (0.21)		
Attention-deficit hyperactivity problems												
No	479/513 (93.37)	0.47 (0.26)	.30	0.21	128/140 (91.43)	0.61 (0.26)	.66	0.06	351/373 (94.10)	0.42 (0.24)	.29	0.21
Yes	34/513 (6.63)	0.41 (0.25)			12/140 (8.57)	0.60 (0.17)			22/373 (5.90)	0.32 (0.23)		
Conduct problems												
No	476/512 (92.97)	0.47 (0.26)	.09	0.27	123/140 (87.86)	0.62 (0.25)	.08	0.45	353/372 (94.89)	0.42 (0.25)	.09	0.27
Yes	36/512 (7.03)	0.40 (0.24)			17/140 (12.14)	0.51 (0.26)			19/372 (5.11)	0.30 (0.18)		
Oppositional defiant problems												
No	465/513 (90.64)	0.47 (0.26)	.02	0.34	126/140 (90.00)	0.62 (0.25)	.20	0.34	339/373 (90.88)	0.42 (0.24)	.02	0.40
Yes	48/513 (9.36)	0.38 (0.23)			14/140 (10.00)	0.53 (0.22)			34/373 (9.12)	0.32 (0.21)		
Somatic problems												
No	443/513 (86.35)	0.48 (0.26)	<.001	0.65	129/140 (92.14)	0.62 (0.25)	.01	0.75	314/373 (84.18)	0.43 (0.24)	<.001	0.55
Yes	70/513 (13.65)	0.32 (0.23)			11/140 (78.57)	0.44 (0.15)			59/373 (15.82)	0.30 (0.24)		
**Social factors**												
Social transition												
Not transitioned	26/454 (5.73)	0.53 (0.25)	.03^i^	0.02	9/87 (10.34)	0.55 (0.27)	.72^j^	0.01	17/367 (4.63)	0.52 (0.25)	.06^k^	0.02
Partially transitioned	244/454 (53.74)	0.45 (0.24)			47/87 (54.02)	0.57 (0.24)			197/367 (53.68)	0.42 (0.24)		
Fully transitioned	184/454 (40.53)	0.41 (0.25)			31/87 (35.63)	0.52 (0.28)			153/367 (41.69)	0.38 (0.24)		
Support for gender identity												
Support from at least 1 parent												
Not supportive	16/504 (3.17)	0.41 (0.29)	.40	−0.24	2/138 (1.45)	0.76 (0.34)	.37	0.60	14/366 (3.83)	0.36 (0.26)	.48	−0.24
Fully supportive	488/504 (96.83)	0.47 (0.26)			136/138 (98.55)	0.61 (0.25)			352/366 (96.17)	0.42 (0.24)		
Support from mother												
Not supportive	28/498 (5.62)	0.42 (0.27)	.32	−0.20	3/137 (2.19)	0.59 (0.38)	.92	−0.07	25/361 (6.93)	0.40 (0.25)	.83	−0.07
Fully supportive	470/498 (94.38)	0.47 (0.26)			134/137 (97.81)	0.61 (0.25)			336/361 (93.07)	0.41 (0.24)		
Support from father												
Not supportive	80/413 (19.37)	0.45 (0.28)	.29	−0.12	22/121 (18.18)	0.65 (0.27)	.69	0.15	58/292 (19.86)	0.38 (0.25)	.14	−0.22
Fully supportive	333/413 (80.63)	0.49 (0.26)			99/121 (81.81)	0.61 (0.25)			234/292 (80.14)	0.43 (0.25)		
Support from siblings												
Not supportive	58/394 (14.72)	0.41 (0.26)	.04	−0.31	21/111 (18.92)	0.48 (0.27)	.009	−0.71	37/283 (13.07)	0.36 (0.24)	.21	−0.25
Fully supportive	336/394 (85.28)	0.49 (0.27)			90/111 (81.08)	0.65 (0.24)			246/283 (86.93)	0.43 (0.25)		
Support from friends												
Not supportive	21/443 (4.74)	0.43 (0.29)	.66	−0.12	11/115 (9.57)	0.49 (0.29)	.15	−0.49	10/328 (3.05)	0.37 (0.29)	.72	−0.17
Fully supportive	422/443 (95.29)	0.46 (0.26)			104/115 (90.43)	0.62 (0.26)			318/328 (96.95)	0.42 (0.24)		
Support from teachers												
Not supportive	19/354 (5.37)	0.42 (0.29)	.58	−0.15	7/101 (6.93)	0.56 (0.27)	.71	−0.16	12/253 (4.74)	0.34 (0.29)	.38	−0.27
Fully supportive	335/354 (94.63)	0.46 (0.26)			94/101 (93.07)	0.60 (0.26)			241/253 (95.26)	0.41 (0.24)		
Gatehouse Bullying Scale^l^												
Not bullied	238/487 (48.87)	0.52 (0.25)	<.001	0.56	49/104 (47.12)	0.64 (0.25)	.02	0.46	189/383 (49.35)	0.48 (0.24)	<.001	0.63
Bullied	249/487 (51.13)	0.38 (0.24)			55/104 (52.88)	0.52 (0.25)			194/383 (50.65)	0.34 (0.22)		

Abbreviations: CHU-9D, Child Health Utility 9D instrument; NA, not applicable.

^a^ Numbers may not sum to the expected totals due to missing data or the respondent preferring not to answer certain questions.

^b^ Effect sizes based on Cohen *d*. For comparisons with more than 2 groups, the partial η^2^ is reported.

^c^ Dunn test: nonbinary vs transgender masculine: *P* = .49; nonbinary vs transgender feminine: *P* = .03; nonbinary vs unsure: *P* = .09; transgender masculine vs transgender feminine: *P* = .006; transgender masculine vs unsure: *P* = .048; transgender feminine vs unsure: *P* = .34.

^d^ Dunn test: nonbinary vs transgender masculine: *P* = .20; nonbinary vs transgender feminine: *P* = .26; nonbinary vs unsure: *P* = .26; transgender masculine vs transgender feminine: *P* = .43; transgender masculine vs unsure: *P* = .46; transgender feminine vs unsure: *P* = .49.

^e^ Dunn test: nonbinary vs transgender masculine: *P* = .47; nonbinary vs transgender feminine: *P* = .04; nonbinary vs unsure: *P* = .23; transgender masculine vs transgender feminine: *P* = .01; transgender masculine vs unsure: *P* = .20; transgender feminine vs unsure: *P* = .19.

^f^ A total score of 28 or more is indicative of clinically significant gender dysphoria. Only those aged 11 years or older completed the Gender Preoccupation and Stability Questionnaire (n = 408).

^g^ Only those aged 11 years or older completed the Columbia Suicide Severity Rating scale (n = 387).

^h^ Based on the Child Behavior Checklist.

^i^ Dunn test: not transitioned vs partially transitioned: *P* = .06; not transitioned vs fully transitioned: *P* = .009; partially transitioned vs fully transitioned: *P* = .04.

^j^ Dunn test: not transitioned vs partially transitioned: *P* = .37; not transitioned vs fully transitioned: *P* = .43; partially transitioned vs fully transitioned: *P* = .21.

^k^ Dunn test: not transitioned vs partially transitioned: *P* = .04; not transitioned vs fully transitioned: *P* = .01; partially transitioned vs fully transitioned: *P* = .10.

^l^ Only those aged 8 years or older completed the Gatehouse Bullying Scale (n = 487).

### QOL Comparison With Population-Based Data

CHU-9D scores for the TGD cohort were compared with Australian population norms (Table 3), and mean (SD) CHU-9D scores were significantly lower compared with population norms both for TGD children (0.58 [0.27] vs 0.81 [0.16]; *P* < .001) and for TGD adolescents (0.41 [0.25] vs 0.80 [0.14]; *P* < .001). We also undertook a more detailed comparison with population-based data from the YMM survey, which specifically examined QOL in adolescents with different mental health diagnoses.^40^ Notably, TGD adolescents who had gender dysphoria but no other mental health problems based on the CBCL had substantially lower mean (SD) CHU-9D scores (0.51 [0.23]) than did those from the YMM cohort with common mental health problems, such as anxiety (0.70 [0.24]) and depression (0.64 [0.26]).

**Table 3.** CHU-9D Scores in the Trans20 Cohort Compared With Age-Matched Australian Population Norms^a^

Age cohort	Trans20 cohort		Australian population norm			Effect size
	No.	CHU-9D score, mean (SD)	No.	CHU-9D score, mean (SD)	*P* value	
**Longitudinal Study of Australian Children, age 11-12 y** ^39^						
Total	56	0.58 (0.27)	1827	0.81 (0.16)	<.001	−1.40
**Community-based adolescents, age 11-17 y** ^24^						
Total	439	0.43 (0.25)	2020	0.82 (0.13)	<.001	−2.43
Age						
≤14 y	202	0.45 (0.25)	1001	0.83 (0.12)	<.001	−2.48
≥15 y	237	0.41 (0.25)	1019	0.80 (0.14)	<.001	−2.41
**Young Minds Matter, age 11-17 y** ^40^						
Total	439	0.43 (0.25)	2967	0.78 (0.20)	<.001	−1.69
Mental health problems including gender dysphoria^b^						
No	36	0.64 (0.26)	1302	0.82 (0.17)	<.001	−1.04
Yes	403	0.41 (0.24)	228	0.74 (0.20)	<.001	−1.46
Gender dysphoria only, no mental health problems based on CBCL^b^						
No	227	0.34 (0.23)	NA	NA	NA	NA
Yes	171	0.51 (0.23)	NA	NA	NA	NA
Attention-deficit/hyperactivity problems^c^						
No	405	0.44 (0.25)	2807	0.79 (0.20)	<.001	−1.69
Yes	24	0.35 (0.24)	160	0.74 (0.19)	<.001	−1.98
Depressive problems^c^						
No	233	0.52 (0.24)	2808	0.79 (0.19)	<.001	−1.39
Yes	196	0.33 (0.22)	159	0.64 (0.26)	<.001	−1.30
Anxiety problems^c^						
No	280	0.48 (0.25)	2870	0.79 (0.19)	<.001	−1.58
Yes	149	0.34 (0.27)	97	0.70 (0.24)	<.001	−1.39
Conduct problems^c^						
No	405	0.44 (0.25)	2919	0.78 (0.20)	<.001	−1.64
Yes	23	0.37 (0.26)	48	0.71 (0.23)	<.001	−1.42

Abbreviations: CBCL, Child Behavior Checklist; CHU-9D, Child Health Utility 9D instrument; NA, not applicable.

^a^ The Trans20 sample was matched to the respective age range reported in the population norms.

^b^ Mental health problems for the Trans20 sample included depressive problems, anxiety problems, attention-deficit/hyperactivity problems, oppositional defiant problems, conduct problems, and somatic problems (all of which were based on the CBCL), while gender dysphoria was based on the Gender Preoccupation and Stability Questionnaire.

^c^ Mental health classification group may include comorbid mental health problems.

### Association Between Gender Dysphoria and QOL

The regression analyses evaluating the association between gender dysphoria and QOL demonstrated that gender dysphoria was an independent factor associated with QOL. It was associated with a 0.152 decrease in the CHU-9D score (β, −0.152; 95% CI, −0.257 to −0.048; *P* = .004) (Table 4).

**Table 4.** Regression Analysis Results

Variable	CHU-9D score (N = 398)^a^	
	β (SE) [95% CI]	*P* value
Constant	0.786 (0.155) [0.481 to 1.090]	NA
Age	0.005 (0.008) [−0.011 to 0.022]	.56
Female gender presumed at birth		
Yes	−0.066 (0.027) [−0.118 to −0.014]	.01
No	1 [Reference]	
Gender dysphoria		
Yes	−0.152 (0.053) [−0.257 to −0.048]	.004
No	1 [Reference]	
Mental health condition		
Yes	−0.181 (0.023) [−0.225 to −0.136]	<.001
No	1 [Reference]	

Abbreviations: CHU-9D, Child Health Utility 9D instrument; NA, not applicable.

^a^ *R*^2^, 0.1817; adjusted *R*^2^, 0.1734; Akaike information criterion, −60.24; bayesian information criterion, −40.31.

## Discussion

This study explored the QOL of TGD children and adolescents at the time of their initial clinical presentation to a large specialist pediatric gender service in Australia. To do so, we used a sample size that was more than 3.5-fold greater than that in, to our knowledge, the largest previously reported study of QOL among TGD young people^13,16^ and a data set that included a comprehensive profile of various demographic, social, and clinical characteristics. We were able to make several important observations.

First, we identified several demographic, social, and clinical characteristics associated with reduced QOL in TGD children and adolescents. In terms of demographics, TGD adolescents had worse QOL compared with TGD children, as did those assigned female at birth. To our knowledge, this is the first time such observations have been reported. While our study was unable to examine the reasons for this finding, unwanted pubertal development is often a crucial trigger for increased gender dysphoria, and this might be expected to reduce QOL during adolescence. At the same time, adolescence is a critical developmental period with increased social demands and during which mental health problems frequently emerge.^43^ For TGD individuals assigned female at birth, reasons for a worse QOL may reflect a higher rate of mental health problems, which we observed to be associated with lower CHU-9D scores in our regression analysis and which have previously been reported by others to occur more commonly in both individuals assigned female at birth from the general adolescent population^44^ and those who are transgender.^45^ With regard to social factors, bullying was associated with significantly reduced QOL scores in TGD young people, which highlights the importance of addressing this harmful behavior. We also noted lower QOL scores in those reporting a lack of support for their gender identity from family, friends, and school, which is in keeping with previous literature regarding support and mental health of TGD young people.^15,46,47^ However, with the exception of support from siblings, the differences that we observed were not statistically significant. This is likely due to the limited power of our sample, which comprised only a small number of young people reporting a lack of support (this is probably a reflection of a sampling bias inherent to clinical cohorts). An unexpected observation in our study was that those who had fully socially transitioned reported lower QOL scores. While this difference was only statistically significant in those aged 13 to 17 years, it is perhaps a reflection that undertaking a social transition can be challenging and stressful for young people, especially for those who have yet to access the support of specialist gender services, as was the case with the present sample. Consistent with this reasoning, a recent study found that social transition during adolescence was associated with greater odds of adverse mental health outcomes driven by unaccepting school environments and experiences of harassment.^47^ In terms of clinical characteristics, we also observed that TGD young people with mental or physical health problems had significantly worse QOL. This finding emphasizes the importance of adequately addressing these issues within this population already at risk of poor QOL.

Second, our results were consistent with observations from previous studies reporting that TGD young people had a lower QOL score compared with general population norms.^13,14,16^ A strength of our study is that we used the CHU-9D, which is a generic pediatric measure that allowed us to compare our data with population norms that are usually not available for specific QOL measures. Additionally, the CHU-9D not only measures QOL domains that are important to children and adolescents but also allows generation of quality-adjusted life-years, which could be used for future economic evaluation studies (eg, to assess the impact of treatment).^29^ Moreover, using YMM data, we found that the mean QOL utility score among TGD young people was lower than that among young people in the general population with serious mental health problems, such as depression and anxiety. This finding suggests that TGD young people experience significant QOL decrements beyond those associated with common mental health difficulties alone.

Third, consistent with this last observation, we identified that the presence of gender dysphoria was independently associated with a 0.152 reduction in the QOL utility score when controlling for other factors. Given that a change in health utility scores of 0.03 is generally considered clinically important for other preference-based measures (no formal minimally important difference is currently available for the CHU-9D),^48^ a reduction of 0.152 is thus substantial and highlights the importance of providing relevant supports and services to help TGD young people better manage gender dysphoria.

### Limitations

This study has limitations, several of which have already been discussed. For instance, we could not compare QOL of TGD children aged 10 years or younger in the sample with age-matched population norms given a scarcity of population-based studies in this age group. It is also important to note that our data were cross-sectional and drawn from participants just prior to their first appointment at a specialist gender service. Given that the Trans20 study is now following up these same young people longitudinally, it will therefore be critical to continue to assess QOL over subsequent data waves; this should enable us to move beyond identifying associations and instead make causal inferences. In terms of the generalizability of our study findings, it is important to note that our study sample represented a gender clinic–referred cohort of TGD young people that represents only a small proportion of the entire TGD youth population; this is an important consideration given differences in nonclinical vs clinical samples.^49,50^

## Conclusions

This cohort study found that the QOL of TGD children and adolescents was worse than that of not only age-matched peers from the general population but also adolescents with serious mental health conditions, such as anxiety and depression. These findings have important public health implications insofar as they suggest that there is a substantial health burden for TGD young people. Our results also suggest multiple areas that can be targeted to improve QOL among TGD young people, including increased efforts to address bullying, coexisting mental and physical health problems, and gender dysphoria. Our findings thus add to the expanding evidence base supporting improved social supports and clinical services for TGD young people.

## Notes

Supplement 1. **eTable.** Summary of the Instruments Used in the Trans20 Study **eFigure 1.** CHU-9D Scores by Age Group **eFigure 2.** CHU-9D Scores by Gender Presumed at Birth **eFigure 3.** Centile Chart Displaying CHU-9D Scores by Age and Gender Presumed at Birth

Supplement 2.
Data Sharing Statement
