# Error in Table 2

**DOI:** 10.1001/jamanetworkopen.2023.5486

**Published:** 2023-03-13

In the Original Investigation titled “Assessment of Quality of Life of Transgender and Gender-Diverse Children and Adolescents in Melbourne, Australia, 2017-2020,”^1^ published February 2, 2023, in Table 2, the stub column labels under “Suicidality” were transposed. The top label should be “Not present,” and the bottom label should be “Present.” This article has been corrected.^1^
