# Supplementary material for: Assessment of Quality of Life of Transgender and Gender-Diverse Children and Adolescents in Melbourne, Australia, 2017-2020
Source: JAMA Netw Open. 2023 Feb 2;6(2):e2254292. doi: 10.1001/jamanetworkopen.2022.54292 (PMC9896293; doi:10.1001/jamanetworkopen.2022.54292)
Supplement: Supplement 1. — eTable. Summary of the Instruments Used in the Trans20 Study eFigure 1. CHU-9D Scores by Age Group eFigure 2. CHU-9D Scores by Gender Presumed at Birth eFigure 3. Centile Chart Displaying CHU-9D Scores by Age and Gender Presumed at Birth [file jamanetwopen-e2254292-s001.pdf]

Supplementary Online Content

Engel L, Majmudar I, Mihalopoulos C, Tollit MA, Pang KC. Assessment of quality of life of transgender and gender-diverse children and adolescents in Melbourne, Australia, 2017-2020. *JAMA Netw Open*. 2023;6(2):e2254292. doi:10.1001/jamanetworkopen.2022.54292

eTable 1. Summary of the Instruments Used in the Trans20 Study

eFigure 1. CHU-9D Scores by Age Group

eFigure 2. CHU-9D Scores by Gender Presumed at Birth

eFigure 3. Centile Chart Displaying CHU-9D Scores by Age and Gender Presumed at Birth

This supplementary material has been provided by the authors to give readers additional information about their work.

© 2023 Engel L et al. *JAMA Network Open*.

eTable 1: Summary of the Instruments Used in the Trans20 Study

**Construct**	**Instrument**	**Age range and source**
**Quality of Life**	Child Health Utility (CHU-9D)	Young person report: 6yrs+
**Demographics**	Trans20 questionnaire (age, sex assigned at birth, education level, country of origin and language spoken at home, postcode)	Parent report: all ages<br>Young person report: all ages
**Gender Identity**	Trans20 questionnaire	Young person report: all ages
**Health information**		
Gender Dysphoria	Gender Preoccupation and Stability Questionnaire (GPSQ)	Young person report: 11yrs+
Physical and mental health problems	Trans20 questionnaire	Parent report: all ages
Mental health problems	Child Behaviour Checklist (CBCL)	Parent report: all ages
Suicidality	Columbia Suicide Severity Rating (CSSR)	Young person report: 12yrs+
**Social factors**		
Bullying	Gatehouse bullying scale	Young person report: 8yrs+
Social Transition	Trans20 questionnaire	Young person report: 8yrs+
Support	Trans20 questionnaire	Young person report: all ages

© 2023 Engel L et al. *JAMA Network Open*.

eFigure 1: CHU-9D Scores by Age Group

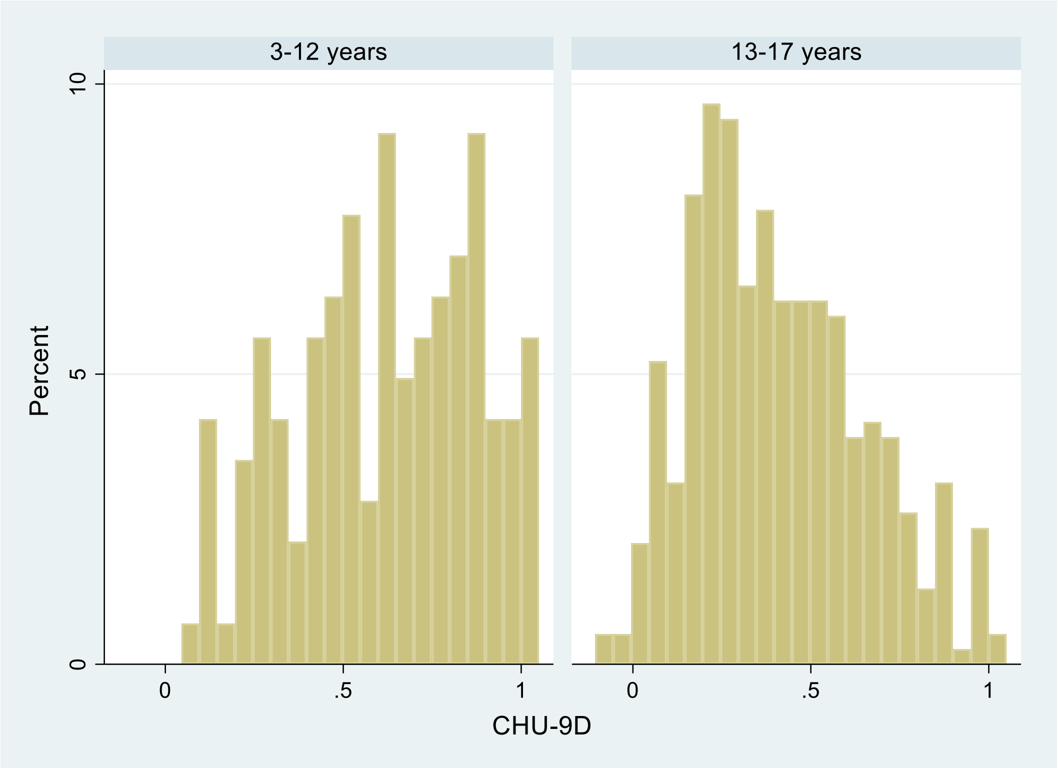

© 2023 Engel L et al. *JAMA Network Open*.

eFigure 2: CHU-9D Scores by Gender Presumed at Birth

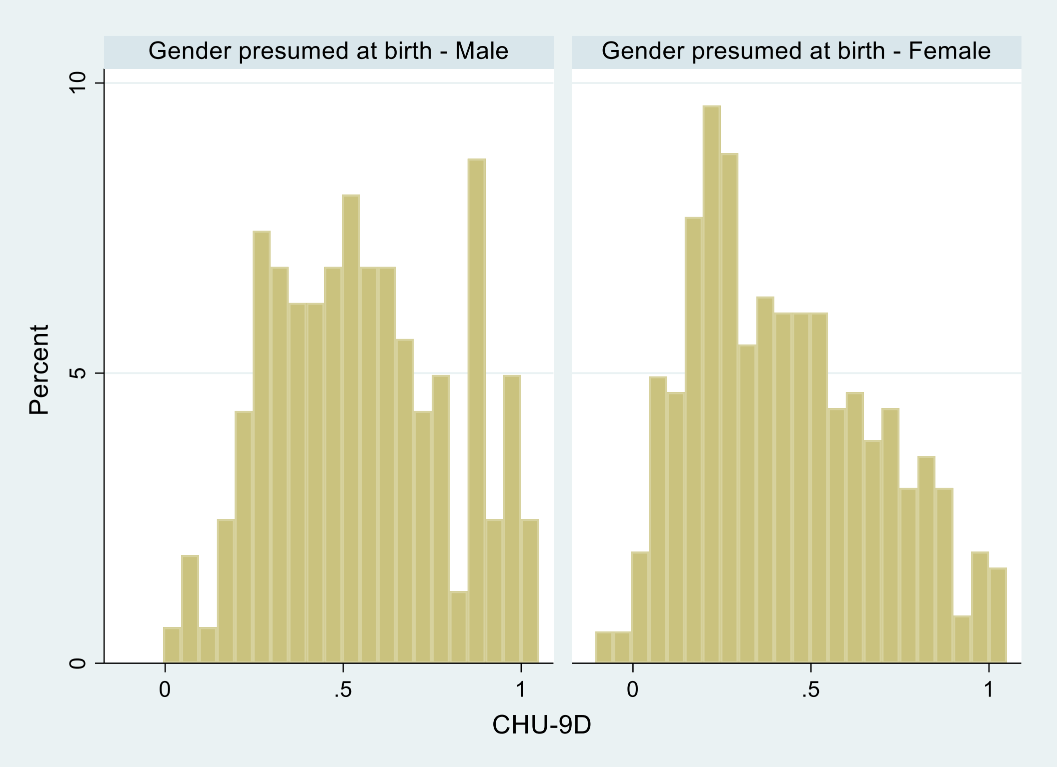

© 2023 Engel L et al. *JAMA Network Open*.

eFigure 3: Centile Chart Displaying CHU-9D Scores by Age and Gender Presumed at Birth

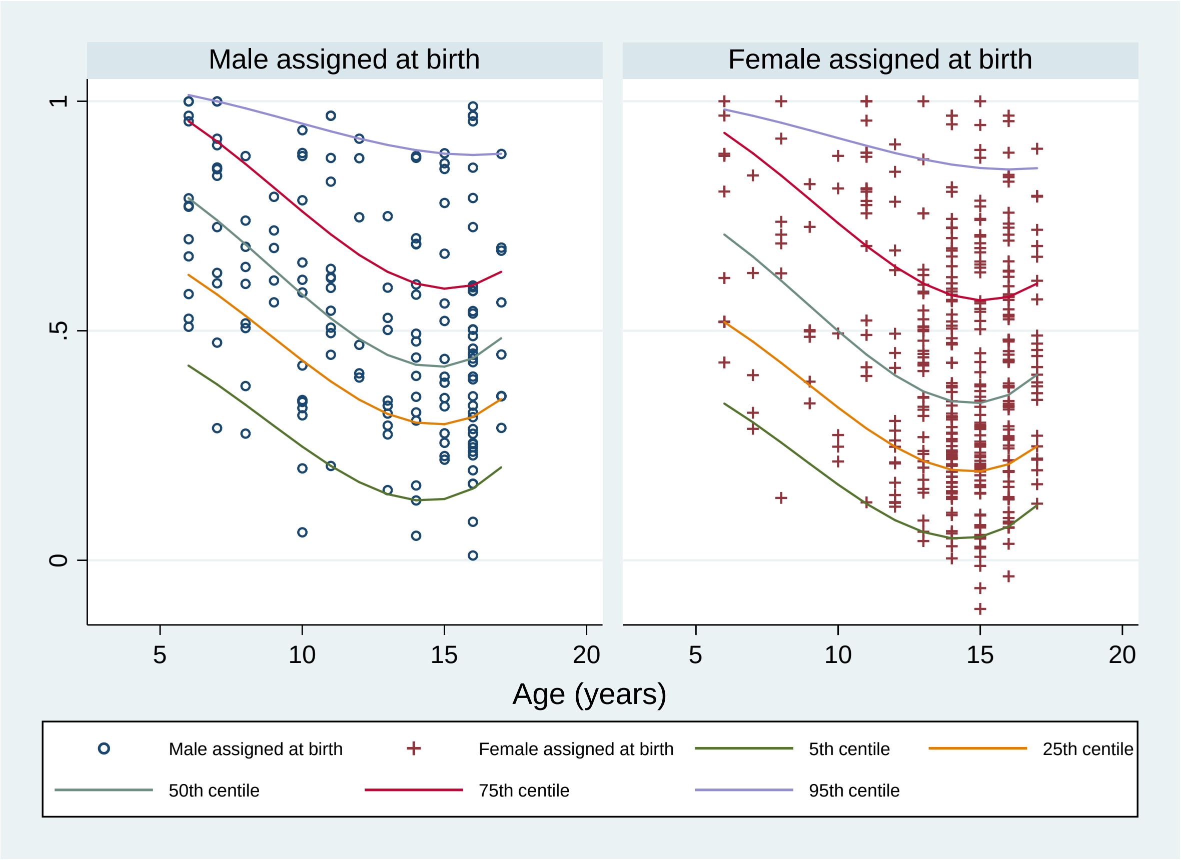

CHU-9D scores are plotted by age and gender presumed at birth. Centiles were predicted using quantile regression for the best fitting model for the mean, where the covariates are the power variable of age and gender presumed at birth.

© 2023 Engel L et al. *JAMA Network Open*.
