# Supplementary material for: Assessment of Quality of Life of Transgender and Gender-Diverse Children and Adolescents in Melbourne, Australia, 2017-2020
Source: JAMA Netw Open. 2023 Feb 2;6(2):e2254292. doi: 10.1001/jamanetworkopen.2022.54292 (PMC9896293; doi:10.1001/jamanetworkopen.2022.54292)
Supplement: Supplement 2. — Data Sharing Statement [file jamanetwopen-e2254292-s002.pdf]

# Data Sharing Statement

Engel. Assessment of Quality of Life of Transgender and Gender-Diverse Children and Adolescents in Melbourne, Australia, 2017-2020. *JAMA Netw Open*. Published February 02, 2023. doi:10.1001/jamanetworkopen.2022.54292

**Data**
**Data available:** No
